# Robotic Roux-en-Y gastric bypass: surgical technique and short-term experience from 329 cases

**DOI:** 10.1590/0100-6991e-20212982

**Published:** 2021-11-19

**Authors:** ANDRE LUIZ GIOIA MORRELL, ALEXANDER CHARLES MORRELL-JUNIOR, ALLAN GIOIA MORRELL, JOSE MAURICIO FREITAS MENDES, ALEXANDER CHARLES MORRELL

**Affiliations:** 1 - Instituto Morrell, Cirurgia do Aparelho Digestivo Robótica e Minimamente Invasiva - São Paulo - SP - Brasil; 2 - Sociedade Beneficente Israelita Brasileira Albert Einstein, Cirurgia Geral e do Aparelho Digestivo Minimamente Invasiva e Robótica - São Paulo - SP - Brasil; 3 - Rede D’Or São Luiz, Cirurgia do Aparelho Digestivo Robótica e Minimamente Invasiva - São Paulo - SP - Brasil; 4 - Hospital Vila Nova Star, Cirurgia do Aparelho Digestivo Robótica e Minimamente Invasiva - São Paulo - SP - Brasil; 5 - Grupo Leforte, Cirurgia do Aparelho Digestivo, Bariátrica e Metabólica Robótica - São Paulo - SP - Brasil

**Keywords:** Obesity, Bariatric Surgery, Metabolic Syndrome, Robotic Surgical Procedures, General Surgery, Obesidade Mórbida, Cirurgia Bariátrica, Síndrome Metabólica, Robótica, Cirurgia Geral

## Abstract

**Objective:**

minimally invasive bariatric surgery is clearly superior over open procedures including better early outcomes. Different surgical approaches are used to treat the severely obese, having Roux-en-Y gastric bypass (RYGB) being a highly frequent procedure. Robotic surgery overcomes some laparoscopic limitations adding ergonomics, articulating instruments and a three-dimensional high definition camera. Based on our vast robotic experience, we present our referred group case series and a standardized Robotic Roux-en-Y gastric bypass (rRYGB) technique as well as its outcomes.

**Methods:**

a review of a prospective maintained database was conducted in patients submitted to robotic Roux en Y bariatric surgery between April 2015 and July 2019. Surgical technique is described and illustrated. We also reported patients demographics, outcomes and its follow-up.

**Results:**

a Retrospective analysis identified 329 patients submitted to Robotic Roux-en-Y gastric bypass. Both da Vinci Si and Xi platforms were used. Mean age was 34.4 years, with median BMI of 44.2 kg/m^2^. Mean console time was 102 min and there was no conversion. No surgical hospital readmission rates were seen in the first 30 days.

**Conclusion:**

this study represents our initial experience of robotic Roux-en-Y gastric bypass (rRYGB), its short outcomes and a standardized surgical technique. Our results encourage that rRYGB is technically feasible and safe, and might offer some advantages showing good outcomes and minimal complications.

## RESUMO

**Objetivo:**

a cirurgia bariátrica minimamente invasiva é claramente superior aos procedimentos abertos, incluindo melhores resultados iniciais. Diferentes abordagens cirúrgicas são usadas para tratar os gravemente obesos, sendo o bypass gástrico em Y de Roux (RYGB) um procedimento muito frequente. A cirurgia robótica supera algumas limitações laparoscópicas adicionando ergonomia, instrumentos de articulação e uma câmera tridimensional de alta definição. Com base em nossa experiência em cirurgia robótica, apresentamos uma série de casos operados com uma técnica de bypass gástrico em Y de Roux robótico (rRYGB) padronizada, bem como seus resultados.

**Métodos:**

foi realizada uma revisão de um banco de dados prospectivamente de pacientes submetidos à cirurgia robotica de bypass gastrico em Roux en Y entre abril de 2015 e julho de 2019. A técnica cirúrgica é descrita e ilustrada. Também relatamos dados demográficos dos pacientes, resultados e seu acompanhamento.

**Resultados:**

uma análise retrospectiva identificou 329 pacientes submetidos ao bypass gástrico em Y de Roux robótico. Ambas as plataformas da Vinci, Si e Xi foram usadas. A média de idade foi de 34,4 anos, com mediana de IMC de 44,2 kg/m^2^. O tempo médio do console foi de 102 min e não houve conversão. Nenhuma taxa de readmissão hospitalar cirurgica foi observada nos primeiros 30 dias.

**Conclusão:**

este estudo representa a experiência inicial de bypass gástrico em Y-de-Roux robótico (rRYGB), seus resultados iniciais e uma padronização da técnica cirúrgica. Nossos resultados são encorajadores, com uma técnica viável, segura, e potencial benefício ao paciente mostrando bons resultados e mínimas complicações.

## INTRODUCTION

Obesity has reached pandemic levels and currently affects more than 650 million individuals worldwide, representing 13% of the global adult population^1^. Bariatric surgery is a well-established treatment option for morbid obese patients, with many available different surgical approaches, which are currently the only effective and durable therapeutic option for this life-threatening condition^2^ ^,^ ^3^. Gastric bypass was first introduced by the so-called “father of obesity surgery” Dr. Edward E. Mason in 1966, and since then, it has been modified into its current form. 

Although open bariatric procedures may be performed, the wound-related complications such as infection and incisional hernia can be troublesome. The benefits of the minimally invasive surgery for morbid obese patients are obvious, offering reduced wound complications and shorter hospital stays^4^. However, performing bariatric surgery can be technically demanding in many situations with a flat learning curve that might require up to 500 cases^5^. Even high skilled surgeons routinely face challenging situations in laparoscopy due to extremely large patients, large and heavy livers, thick abdominal walls and substantial visceral fat, which interfere in exposure, dissection, and reconstruction. 

Robotic surgery gained popularity providing solutions to the challenges posed by laparoscopy, including ergonomics, a high-definition 3-dimensional camera, tremor filtration, a third surgeon arm and wristed instruments. In the bariatric surgical field, for example, these characteristics are translate into the ability to perform a better traction of a normally thick abdominal wall, relieving the surgeon’s physical efforts to overcome the counterproductive forces, as well as a highly stable camera and better manipulation of the surgical structures. 

The use of robotics in bariatric surgery has been evolving since Cadiere et al.^6^ reported their first case, and it is now disseminated worldwide. However, training robotic surgeons is a highly demanding and expensive task. Still, a main limitation of robotic surgery is the perceived higher cost and set-up time compared with laparoscopy. The purpose of this article is to report our standardized robotic Roux-en-Y gastric bypass (rRYGB) technique offering suggestions for a successful learning curve and safe procedure, as well as highlighting potential solutions for common problems. Also, we present our group case series experience and the early outcomes..

## METHODS

This is a retrospective review of a prospective maintained database of all robotic Roux-en-Y gastric bypass operations performed by our single surgical group between April 2015, and July 2019. No laparoscopic or any other bariatric, primary or revisional, surgery were included in this study. Data were collected including patients’ demographics, intraoperative variables and postoperative outcomes as well as the follow-up period outcomes. Surgical indications were all based in a complete and extensive evaluation of each patient in an interdisciplinary scenario, according to worldwide guidelines and patients’ agreement. COEP 42416021.7.0000.0087

### Operative room Setup and Patient preparation

Whenever performing any robotic operation, a clear path between the surgeon’s console and the patient table must exist. Also, the robotic patient cart and vision cart should be correctly disposed assuring the scrub nurse and the bed assistant comfortable range of movements during the procedure. In our standardized rRYGB technique, the operative room setup is prepared according to the da Vinci’s robot type used: Si or Xi platform. 

When using the Si platform (Intuitive Surgical Inc. Sunnyvale, CA, USA), a cephalic docking is done, having the robotic patient cart close to the head of the patient. On the other hand, in the Xi platform, a more versatile and flexible exoskeleton, a left lateral docking is preferred, allowing complete exposure of the head of the patient to the anesthesiologist and a wide bedside space for the assistant. In both cases, the scrub nurse and bedside assistant stand by the patient’s right side.

Under general anesthesia, patients are positioned supine with legs fastened and arms close to the trunk. Having the arms wide open with the Si robot, this dramatically raises the chances of external collision of robotic and patients arms during the operation. On the contrary, when operating with the Xi platform, arms can also be placed wide open if necessary, although not preferred. Also important in bariatric surgery, patients are often large, therefore when arms are close to the trunk, they should be draped slightly posterior to the hemiaxilar imaginary line or on its limit, but never anteriorly. Positioning the arms, a bit “dropped” also helps to create space for the lateral port mobility. Antibiotic prophylaxis is routinely used with administration of intravenous cefazolin during anesthetic induction. 

### Port placement and instruments

Pneumoperitoneum is carried out with a Veress needle puncture at the Palmer’s point and carbon dioxide insufflation. After the abdominal cavity is inflated with the pneumoperitoneum of 15mmHg CO2, the exact place for port placement is marked. Port placement for the da Vinci Surgical System is based on the concept of the surgical workspace, placing the first port approximately 15-18mm below the xiphoid, which will be the camera port. It’s important to state that some modifications to the port locations may be necessary due to the patient’s anatomy to ensure correct distance to the target anatomy in taller or shorter patients or extremely obese patients.

**Figure 1:**
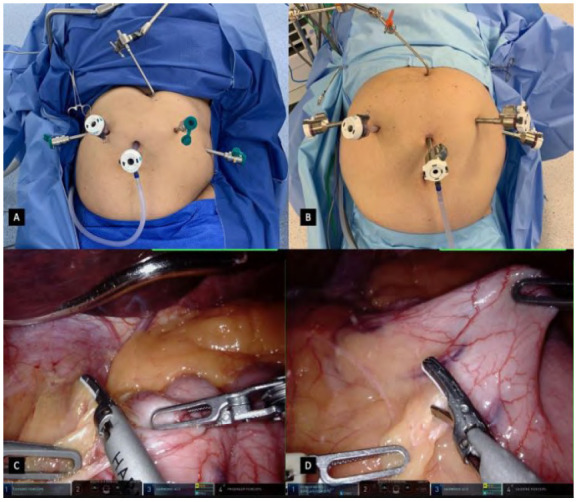
A: Da vinci Si port placement for a rRYGB. B: Da vinci Xi port placement for a rRYGB. C: Hiss angle dissection and visualization of the left crux. D: Lesser omentum opening to achieve the lesser sac and visualization of the posterior wall of the stomach.

Whenever utilizing the da Vinci Si or Xi platform, a 12mm or 8mm camera port is used, and a 30 degree camera is inserted. Then, the patient’s table is placed in a reverse Trendelenburg position without tilt, followed by the direct introduction of the other robotic assistant ports and a Nathanson liver retractor, fixed in the right side of the patient table. The trocars setup is similar in both Si and Xi platforms (Figures 1A/1B); however, the Si requires at least 8cm distance and non-parallel shape to assure minimum collision. 

A robotic port is subsequently placed along the patients right anterior axillary line, above the level of the camera port, which will be the surgeon’s left hand. This port should be as lateral as possible, creating enough distance to the area where the assistant port will be placed. The other 2 robotic 8mm ports are placed in the left anterior axillary line, at the level of the camera port and midway between the camera trocar and this last port, however, cephalad to the camera port line. Those ports are going to be the surgeons’ right hand and robotic assistant arm. Lastly, the 12mm assistant port is placed midway from the camera and left-hand port. Once all ports are ready, the Nathanson liver retractor is positioned in the subxiphoid area and the robotic system is docked. If a Xi platform is used, the camera is first attached to the robotic arm n.2 and targeting the optimal surgical quadrant is done, looking to the gastroesophageal junction (GEJ). 

The procedure is performed using the Harmonic ACE Curved Shears, a fenestrated bipolar, a Cadiere and a megasuturecut needle driver. Instrument positioning is described as Arm 1: Fenestrated bipolar forceps (surgeons left hand); Arm 2: endoscope 30° down; Arm 3: Harmonic ACE Curved Shears and megasuturecut needle driver (surgeons’ right hand, which is temporarily exchanged during operation). Arm 4: Cadiere grasper (surgeons’ right hand).

### Step-by-step robotic Roux-en-Y gastric bypass

#### Cavity inspection

First, the abdominal space is carefully inspected. If present, previous adherences are analyzed to assure a safe pouch creation and the possibility of bringing a jejunal loop long enough to the supramesocolic quadrant for anastomosis. If previous liver to stomach adherences are present, they are first detached and then a liver retractor is positioned.

#### Pouch creation

**Figure 2:**
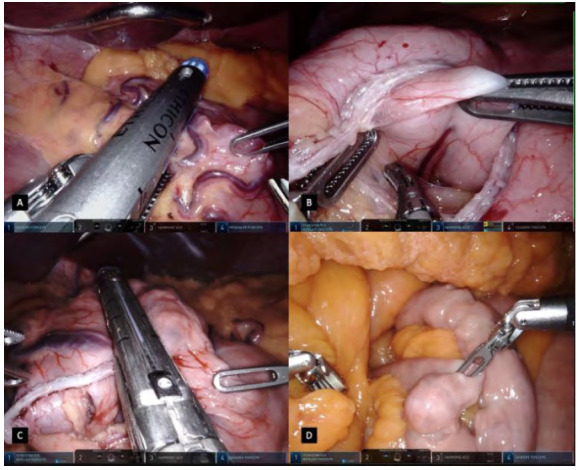
A: A 45mm linear stapler clamped and fired transversely assuring an equal anterior and posterior stomach wall division. B: Clearing posterior stomach wall of any possible adherences. C: Pouch creation with subsequent 45mm linear staplers, calibrated by an oral bougie. D: Identification of Treitz angle and first jejunal branch.

The first step in our standardized rRYGB is creating the pouch and before starting dissection, stomach bougie aspiration by the anesthesiologist is asked for fluid evacuation if needed. Initially, the stomach is pulled caudally and the Hiss angle is dissected to assure an optimal visualization of the left crux (Figure 1C). Then, by pulling the gastric anterior wall laterally, an incision in the lesser omentum is performed 5cm below the GEJ and close to the stomach, to achieve the lesser sac and have a clear visualization of the posterior wall of the stomach (Figure 1D). This step of the procedure is done with extreme finesse, dissecting the fat tissue and cauterizing small vessels to avoid any bleeding. Once this retrogastric tunnel is achieved, a 45mm linear stapler is introduced in the assistant port, clamped and fired transversely assuring an equal anterior and posterior stomach wall division and all surgical staples are fired after 30 seconds of tissue precompression by the bedside assistant (Figure 2A). After the first stapling is done, the posterior stomach wall is cleared of any adherences (Figure 2B) and a second stapling is positioned and fired in a cranial direction, after a 32 french orally inserted bougie calibration has been done. Then, complete exposure of the Hiss angle is achieved and a connection between the anterior and posterior compartments is done. One or two more staplings are fired to complete the pouch creation and disconnection of the excluded stomach (Figure 2C). This last staple is performed not extremely close to the Hiss angle, to avoid damage to the collar sling fibers and the circular muscles around the GEJ. Finally, a small orifice is created in the distal and posterior wall of the gastric pouch using the harmonic scalpel for a gastrojejunal anastomosis. 

### Biliopancreatic limb creation and Gastrojejunal anastomosis

**Figure 3:**
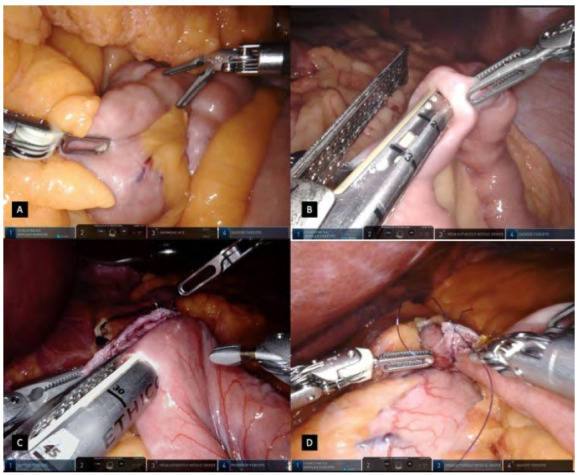
A: Jejunum bowel measured assuring the proximal segments to be laterally in the abdomen. B: A 45mm linear stapler inserted in the jejunum for a gastrojejunal anastomosis. C: A side-to-side gastrojejunostomy fashioned and calibrated having an equal positioning of both tissues. D: Anastomosis single stitch in the medial aspect of the gastrojejunal orifice.

Robotic and assistant arms are used to pull the transverse colon and its mesocolon upwards, until the ligament of Treitz is seen (Figure 2D). Once the first jejunal segment is clearly identified, 100cm of the jejunum is measured assuring the proximal segments to be laterally in the abdomen, to the patient’s left side (Figure 3A). The stated bowel segment is then tractioned by both fenestrated forceps and an enterotomy is done antimesenterically with the Harmonic device. Using the assistant 12mm port, a 45mm linear stapler is inserted in the jejunum, with a partial closure of the stapler, obtaining a better grip (Figure 3B). The transverse colon and greater omentum are lowered to the inferior part of the abdomen and the jejunum is mobilized antecolically to the stomach pouch. 

**Figure 4:**
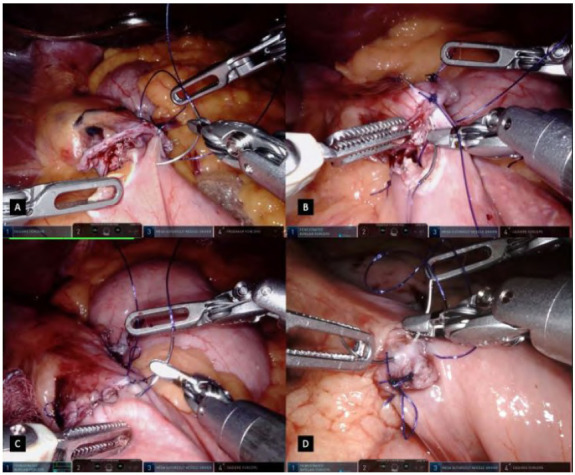
A: First layer of closure in an inverted T extramucosal continuous technique with forehand bites in the jejunum. B: First layer of closure in an inverted T extramucosal continuous technique with backhand bites in the stomach. C/D: A second layer of suture is done in an unidirectional continuous seromuscular technique.

The other blade of the stapler is inserted into the stomach pouch and a side-to-side gastrojejunostomy is fashioned and calibrated in 2-3cm, having an equal positioning of both tissues (Figure 3C). The stapler is fired after a compression time and both branches are opened partially and carefully removed from the lumen to not tear it. Harmonic ACE Curved Shears on the robotic arm n.3 are temporarily exchanged with the megasuturecut needle driver for the gastrojejunostomy conclusion. Before its running suture, a single stitch is done in the medial aspect of the gastrojejunal orifice as a landmark which allows tissue manipulation (Figure 3D). The first layer of closure is fashioned in an inverted T extramucosal continuous technique, having forehand bites in the jejunum and backhand bites in the stomach with absorbable suture (Figures 4A/4B). A second layer of suture is done in a unidirectional continuous seromuscular technique (Figures 4C/4D). During the first suture, after partial closure of the lumen, an oral bougie is manipulated by the anesthesiologist and passed through the anastomosis. During this step of the procedure, the robotic arms n.1 and n.3 are dynamically used for suturing while arm n.4 is used for optimizing the surgical field and the position of structures, based on suture traction and jejunal loop presentation. The sutures are finished off in the medial aspect of the defect, overpassing the medial stitch, ensuring a good anchoring in the tissue. 

### Alimentary limb creation and jejunojejunal anastomosis

**Figure 5:**
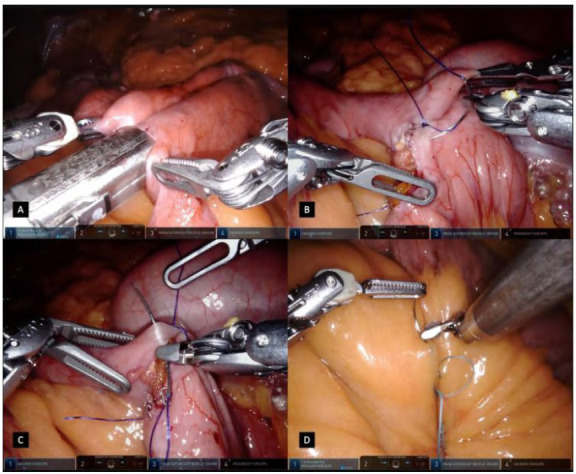
A: A side-to-side jejunojejunostomy created using the 45mm linear stapler. B/C: single extramucosal running suture technique. D: Mesenteric defects closure with a running suture.

Once the gastrojejunostomy is done, the Harmonic ACE Curved Shears are reattached to the robotic arm n.3. An enterotomy is done distancing approximately 5-10cm from the anastomosis, in the antimesenteric border of the jejunum disposed laterally to the pouch, on the patient’s left side. Using the fenestrated forceps and Cadiere grasper, a 100cm alimentary limb is measured distal to the anastomosis and another enterotomy is also created in the antimesenteric border of the jejunum. A side-to-side jejunojejunostomy is created using the 45mm linear stapler with carefully opening to avoid stress on the anastomosis (Figure 5A). The enterotomy is closed in a single extramucosal running suture technique, assuring hermetic and good bowel coaptation, having a simple stitch in the medial border of the anastomosis for its delimitation and tissue manipulation during anastomosis (Figures 5B/5C).

### Mesenteric defect closure and bowel loop transection for a Roux-en-Y anatomy

**Figure 6:**
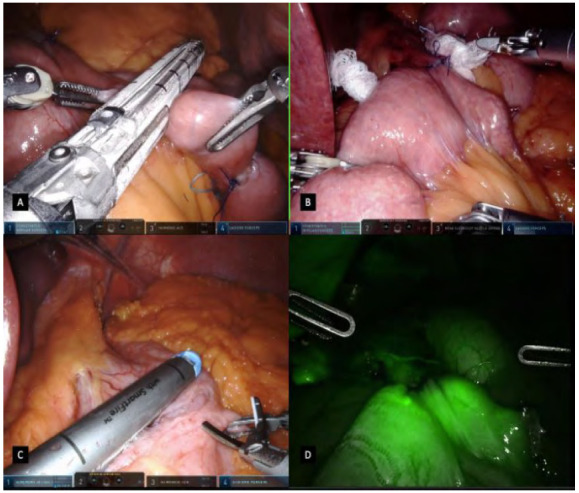
A: Transection of the bowel loop created for the gastrojejunostomy using a 45mm linear stapler, assuring a Roux-en-Y anatomy. B: Gastrojejunal anastomosis methylene blue test. C: Using a Robotic Sureform with SmartfireT stapler for a total robotic RYGB. D: Gastrojejunal anastomosis vascular assessment using ICG with Firefly technologyT.

After completion of the jejunojejunostomy, mesenteric defects are closed to avoid internal hernias with a running suture of Ethibond® 2-0 suture (Figure 5D). This suture is done beginning in the deeper part of the meso running to its superficial part, ending close to the bowels. Finally, the bowel loop created for the gastrojejunostomy is transected using a 45mm linear stapler, detaching the jejunojejunostomy from the gastrojejunostomy, pursuing a small residual candy can and creating the Roux-en-Y anatomy (Figure 6A). 

### Leak Test and drainage

The gastrojejunal anastomosis is tested with methylene blue dye administered through the oral bougie. Two sponge gauzes are placed posteriorly to the anastomosis and laterally to the pouch, close to the stapler line while the alimentary limb is sealed (Figure 6B). If positive, a detailed revision of the anastomosis is made and additional stitches are performed followed by a second test. A silicon Blake drain is placed through the 12mm assistant port and exteriorized on patients’ right upper quadrant, through the left surgeon’s arm trocar by removing the left robotic instrument and undocking the robotic arm. The liver retractor is withdrawn and the trocars are retrieved under direct visualization. 

### Technical variations in the armamentarium

A recent addition to the robotic-assisted instruments is the da Vinci stapling technology. If available, the surgeons may choose to apply robotic staplers during the procedure and some technical aspects must be highlighted (Figure 6C). Robotic staples must be used with 12mm width ports, demanding a strategic port placement design. In the rRYGB, all staplings are fired coming from the right to the left side of the patient. For a more practical and efficient technique, our group standardized a 12mm robotic port upfront in the patient’s right anterior axillary line instead of the 8mm robotic port commonly used for the surgeon’s left hand in arm n.1. By having this port setup, all staples are done through this 12mm port with no additional assistant port or camera and instruments exchange between robotic arms. Also, this technique allows a rRYGB with 5 incisions rather than 6 whenever using the laparoscopic stapler with an assistant port. 

A leak test related and similar to the methylene blue dye and easily visible even if there are minor leaks is the indocyanine green (ICG), which has been shown to be also an excellent agent for leak testing through fluorescence image guided technology. By diluting the 25mg of ICG to 250mL in sterile water or saline, this green stained solution is administered through the oral bougie and the surgeon in the console activates the Firefly™ mode, with greenish leak evident whenever present. Also, not routinely performed but possible, tissue perfusion assessment to help estimate the blood supply of visceral anastomosis is possible using intravenously ICG (Figure 6D).

Another possible instrument variation to be described, due to the prospective discontinuation of the Harmonic ACE Curved Shears by intuitive; is by using a monopolar curved scissor or even its most recent advanced Vessel Sealer Extend or Synchro Seal as energy devices. These instruments allow dissection and coagulation with the endowrist technology. .

## RESULTS

This standardized technique has been formulated and refined by our single surgical group having 329 patients undergoing a robotic Roux-en-Y gastric bypass between April 2015 to July 2019. There were predominantly female patients with 246 cases (74.8%) and 83 males (25.2%). Mean age was 36 years (range: 18-66) and the mean body mass index (BMI) was 44.8 kg/m^2^ (range: 35.8-69.8 kg/m^2^). 

Robotic surgery was performed in all cases, with no conversion to laparoscopic or open technique. Da Vinci Xi platform represented 89 (27.0%) of the procedures while 240 (73.0%) were performed using the Si robotic technology. Although previously described and shown, the robotic staplers for a rRYGB, in this series we only included patients undergoing rRYGB technique with laparoscopic staplers. Mean console time was 94 minutes (range: 69-172 min) with mean time docking of 3 minutes (range: 1-6 min). Patients were discharged within 48 hours of their stay in the majority of the cases and there was no 30-days mortality rate. No anastomotic leakage, bleeding, surgical site infection, intraoperative complications or patient reoperation occurred. There were no major complications within the short- and long-term follow-up period (range: 48-1980 days).

## DISCUSSION

Bariatric surgery is well established in the treatment of morbid obesity. However, the role of the robotics in bariatric procedures is still unclear. Robotic systems are increasingly being used in surgical practice, offering superior dexterity, an enhanced visualization, and wristed instruments. A variety of surgical procedures have been described showing benefits using the robotic technology, exemplified by the urology and visceral surgery field^7^ ^-^ ^9^. In bariatric surgery, conventional laparoscopy comes with certain technical limitations, which are amplified by frequently higher BMI obese patients. Increased liver size, high volume of visceral fat and thick abdominal walls are challenging situations which aggravate difficulties while handling manual instruments used in laparoscopy. Besides that, potential benefits of robotic surgery could be more apparent in procedures such as the rRYGB, involving multiple suturing tasks and dissection in fatty and narrow spaces. 

Most comparative data between laparoscopic and rRYGB come from observational studies and not from high-quality randomized controlled trials^10^. Complex bariatric surgeries have already been described safely with the robotic system and with low complication rates^11^. The robot seems to be advantageous in superobese and revisional cases, however primary bariatric procedures such as RYGB could also benefit from the technology with lower anastomotic leak rates^10^. Also, regarding surgical education, the learning curve of rRYGB has also been shown to be shorter when compared to laparoscopic RYGB^12^. 

Even though robotics have been introduced to the surgeons’ armamentarium in the early 2000s, its widespread still faces some difficulties especially in developing countries. Currently, higher costs hamper a wider use of the robotic technology in most healthcare systems. The costs of a robotic operation can be easily divided into categories such: initial purchase, maintenance and disposable parts, which initially may appear to be prohibitive. Still, implementation of robot-assisted operations requires an interdisciplinary approach, with appropriate training of surgical, anesthetic, nurse and technical staff, besides a proctoring program. As the demand for technology and innovation in humanity increases, so does its costs initially. It has been discussed to what extent the additional costs are compensated by lowering and avoiding complications. We believe that capital costs would be amortized over time having a long-term better healthcare assistance with the advent of new robotic platforms by companies across the world, stimulating the market and decreasing technology overall costs^13^. 

As important as the technology itself, to proctor and capacitate the surgeons to a safe robotic assisted-surgery is mandatory. Non-experienced surgeons’ learning curve may be lowered by experts’ robotic-surgeons’ tips and tricks. Previous studies that analyzed the learning curve of laparoscopic gastric bypass suggested that a safe and effective procedure was achieved after having approximately a hundred cases done by the surgeon^14^. The Morrell Institute, as a high volume robotic surgery group in Brazil, believe that by using the robotic platform and a standardized technique, it is possible to shorten the learning curve especially when performing delicate and precise maneuvers with such fine dissections and suturing, that are mandatory during RYGB surgery. 

The present study has focused more on a standardized technique instructing a step-by-step guide to a rRYGB rather than a comparison to bariatric procedures. We believe this report may elucidate and bring robotic surgeon trainees more safety and familiarity whenever performing a rRYGB. Our case series reported no surgical mortality or procedure related complications, showing feasibility as well as good postoperative outcomes. However, some known study limitations related to its intrinsic retrospective analysis are present. Although robotic surgery continues to increase in popularity, larger single-center or multicenter experiences are necessary and will continue to help clarify the role of robotics in primary and also revisional bariatric surgery. 

## CONCLUSION

This study represents our initial experience of robotic Roux-en-Y-gastric bypass and a standardized technique. In this retrospective analysis, our group shows that a standardized rRYGB technique using robotic technology in experienced surgeons’ hands allows a safe procedure and shows encouraging outcomes. Digital platforms are promising in the surgical field although they still require prospective randomized trials to access its real benefits.
